# Biofilm disruption by an air bubble reveals heterogeneous age-dependent detachment patterns dictated by initial extracellular matrix distribution

**DOI:** 10.1038/s41522-017-0014-5

**Published:** 2017-03-03

**Authors:** Hongchul Jang, Roberto Rusconi, Roman Stocker

**Affiliations:** 1 Ralph M. Parsons Laboratory, Department of Civil & Environmental Engineering, Massachusetts Institute of Technology, Cambridge, MA 02139 USA; 2 Department of Civil, Environmental and Geomatic Engineering Institute for Environmental Engineering, ETH Zurich Zurich, 8093 Switzerland; 3 Department of Biomedical Sciences, Humanitas University, Milan, 20089 Italy

## Abstract

Bacteria often adhere to surfaces, where they form communities known as biofilms. Recently, it has been shown that biofilm formation initiates with the microscopically heterogeneous deposition of a skeleton of extracellular polymeric substances (EPS) by individual cells crawling on the surface, followed by growth of the biofilm into a surface-covering continuum. Here we report microfluidic experiments with *Pseudomonas aeruginosa* biofilms showing that their “hidden” heterogeneity can affect the later dynamics of their disruption. Using controlled air bubbles as a model for mechanical insult, we demonstrate that biofilm disruption is strongly dependent on biofilm age, and that disruption to early-stage biofilms can take the shape of a semi-regular pattern of ~15 µm diameter holes from which bacteria have been removed. We explain hole formation in terms of the rupture and retreat of the thin liquid layer created by the long bubble, which scrapes bacteria off the surface and rearranges their distribution. We find that the resulting pattern correlates with the spatial distribution of EPS: holes form where there is less EPS, whereas regions with more EPS act as strongholds against the scraping liquid front. These results show that heterogeneity in the microscale EPS skeleton of biofilms has profound consequences for later dynamics, including disruption. Because few attached cells suffice to regrow a biofilm, these results point to the importance of considering microscale heterogeneity when designing and assessing the effectiveness of biofilm removal strategies by mechanical forces.

## Biofilm disruption: patterns of weak spots

Using air bubbles to disrupt biofilms reveals patterns in biofilm structure and susceptibility to damage that depend on a biofilm’s age. Roman Stocker and colleagues at the Swiss Federal Institute of Technology in Zurich (ETH) used controlled flow of an air bubble to understand mechanical disruption of biofilms of *Pseudomonas aeruginosa*. This physical assault caused a characteristic pattern of holes in early-stage biofilms, different from the damage pattern in older biofilms. The holes were most likely to occur in regions that were relatively deficient in the polymers released by bacterial cells to form the extracellular structure of the biofilm. These findings highlight the importance of heterogeneities in the structure of biofilms as they form. The researchers suggest more attention should be given to age-related variations in microscale structure when designing strategies to disrupt biofilms.

## Introduction

Biofilms are surface-associated microbial communities encased in a self-secreted matrix of extracellular polymeric substances (EPS). Biofilms account for the largest fraction of bacterial biomass on the planet, and often have deleterious effects in natural, industrial and medical settings.^1^ In the environment, biofilms can mobilize heavy metals such as mercury and arsenic, causing stream or soil contamination.^2^ In industrial processes, the formation of biofilms is responsible for huge economic losses (in the billions of dollars yearly) resulting from biofouling and biocorrosion,^3^ which leads to equipment clogging and damage, and product contamination.^4^ In medicine, biofilms represent the major source of infections associated with catheters and implanted devices.^5^ Despite the importance of finding effective methods for biofilm removal in these and other applications, our understanding of biofilm development and in particular of the mechanisms responsible for biofilm detachment remains far from complete.

Detachment refers to the release of bacterial cells or clusters from the surface of the biofilm into the bulk fluid. Several factors can contribute to detachment, including matrix-degrading enzymes,^6^ nutrient levels,^7^ and quorum-sensing signals.^8^ Mechanical forces associated with fluid flow have also been investigated as potential approaches to remove adsorbed bacteria from surfaces,^9, 10^ with a striking example involving the passage of bubbles or air plugs.^11, 12^ Air bubbles remove bacteria from a surface when the three-phase line (separating the liquid, the air and the solid surface) contacts the cells.^13^ The capillary action of moving air-liquid interfaces is known to cause colloidal aggregation^14^ and to generate forces that tend to detach bacteria from surfaces in a broad range of environments, including in the oral cavity during eating, speaking, drinking and swallowing,^15^ in the eye and on contact lenses during blinking,^16^ and on rocks and ship hulls in aquatic systems.^17^ However, previous research on biofilm detachment by air bubbles has focused on endpoint measurements to quantify the net amount of biofilm removed, whereas the mode of biofilm disruption has remained unexplored.

Here we show that, for early-stage biofilms (when bacterial colonies are organized as monolayers), insult by mechanical forces results in a new phenomenon, whereby the passage of a long bubble opens regular holes in the biofilm but fails to completely remove it. We rationalize this finding in terms of the competition between dislodging shear forces and the spatially varying adhesion strength resulting from intrinsic heterogeneity in EPS distribution within the biofilm.

## Results and discussion

**Fig. 1:**
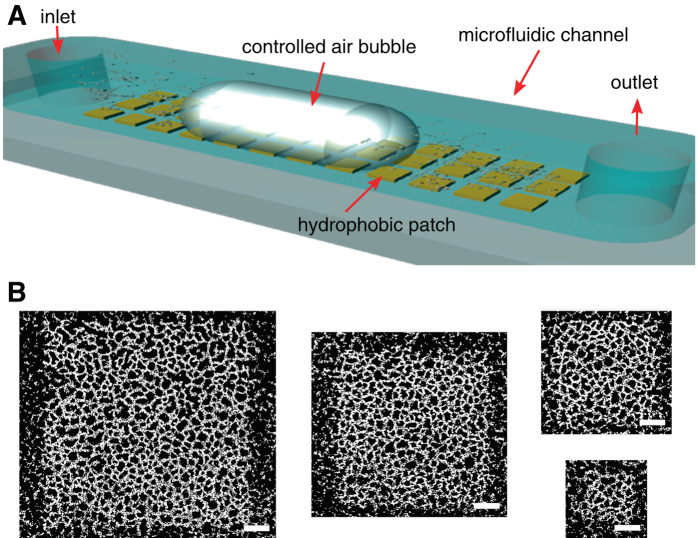
The passage of a long air bubble creates a characteristic pattern of holes in a biofilm. **a** Schematic of the microfluidic setup showing the geometry of the microchannel and the experimental method, in which *P. aeruginosa* bacteria preferentially attached to hydrophobic patches. After a specified growth time (4 h, 8 h, or 12 h), a controlled air bubble was injected in the channel at a mean flow speed of 250 µm s^−1^. **b** Residual biofilm after the passage of the bubble, revealing a semi-regular pattern of holes formed in an 8 h-old biofilm, for different initial patch sizes (measuring 400^2^ μm^2^, 300^2^ μm^2^, 200^2^ μm^2^, and 100^2^ μm^2^). Scale bars, 50 µm

We studied the formation and disruption of controlled *Pseudomonas aeruginosa* PA01 biofilm patches on the glass bottom of a microfluidic channel (Fig. 1a). Biofilm patches formed by the preferential adhesion of *P. aeruginosa* cells to hydrophobic square patches, previously created on the glass by a microcontact printing (Supplementary Figs S1-S2; Methods) technique.^18^ A dilute bacterial suspension (optical density OD_600 _~ 0.2) was injected in the channel and incubated under quiescent conditions for 1 h, allowing cells to attach to the channel’s surfaces. Then, the bacterial suspension was replaced by a minimal culture medium (M63), which was flown continuously at 3 µl min^−1^ (average flow velocity = 250 µm s^−1^) to supply adhering cells with nutrients. Over the course of a few hours, *P. aeruginosa* cells progressively covered the surface of the hydrophobic patches. While some bacteria attached to the surface outside of the patches, most bacteria adhered onto the patches, where cell adhesion was greatly favored by the substrate’s strong hydrophobicity. The concentration of adhering cells could be controlled by varying the concentration of the chemical octadecyltrichlorosilane (OTS; *see* Methods) used in printing the patches.

To determine the effect of a mechanical insult on an early-stage biofilm, the patches were exposed to the controlled passage of a long bubble. The bubble was created by rapidly switching injection from medium to air, creating an approximately 2.5 mm-long bubble traveling at 250 µm s^−1^ (Methods). The air bubble traveled over each patch in approximately 10 s, causing a dramatic and highly characteristic disruption of the original biofilm patch: the resulting biofilms were in the shape of a semi-regular pattern of holes, from which bacteria had been entirely removed, separated by ‘bacterial levees’, consisting of a concentrated monolayer of cells (Fig. 1b). Image analysis showed that holes, which varied in shape but displayed no obvious asymmetry associated with the flow direction, had an equivalent radius of 6.5–8 µm and the surface porosity of the end-state biofilm was ~68%. This pattern was highly consistent among different patches within the same microchannel and among replicate experiments. Experiments with patches of different size (100^2^, 200^2^, 300^2^ and 400^2^ µm^2^) showed that the pattern and its porosity were independent of patch size within the range explored (Supplementary Fig. S3). Moreover, a chemical analysis of the surface using scanning electron microscopy coupled with energy dispersive X-ray spectroscopy (SEM/EDX) showed that the OTS coating was stable even under high shear flows and confirmed that the holes in the biofilm resulted from the detachment of bacteria, rather than the detachment of the underlying OTS layer (Supplementary Fig. S4).

**Fig. 2:**
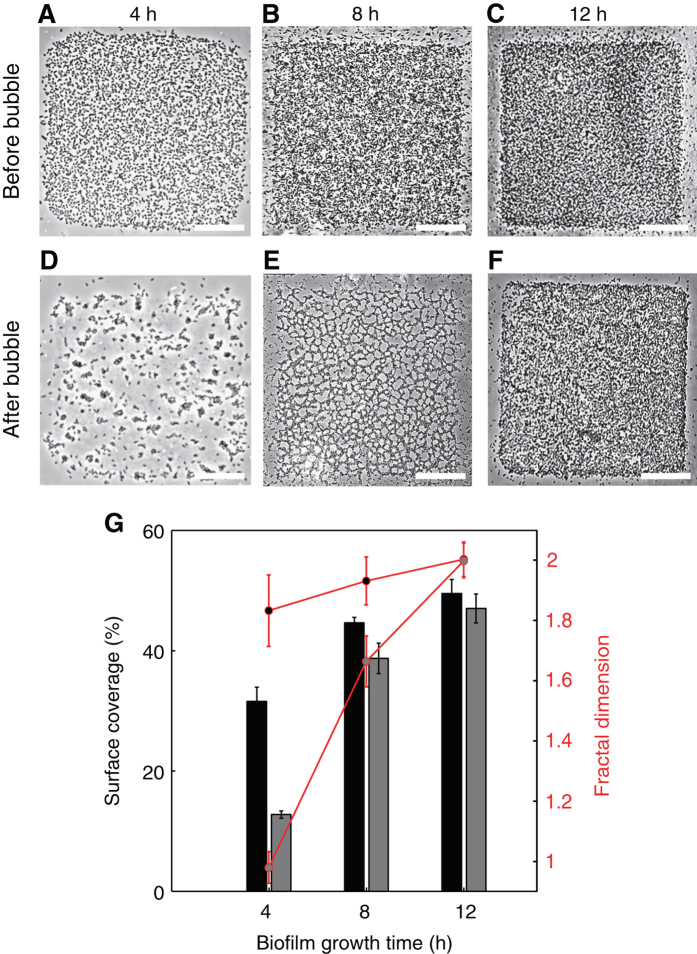
The effect of the air bubble strongly depends on biofilm age. **a**–**f** Biofilms grown for different times (4 h, 8 h, 12 h), shown before (**a**–**c**) and after (**d**–**f**) the passage of a long bubble. The bubble traveled from left to right. Scale bars, 100 µm. **g** Surface coverage (fraction of the surface of a hydrophobic patch covered by bacteria) before (*black*) and after (*gray*) the passage of a bubble, for biofilms of different age (4 h, 8 h, 12 h). The *red curve* (*right axis*) shows the fractal dimension of the cell distribution measured before (*top red curve*) and after (*bottom red curve*) the passage of a bubble. Error bars correspond to the standard error of the mean over five patches

The adhesion properties of an early-stage biofilm are strongly dependent on the biofilm growth time. This is clearly revealed by comparing experiments in which the air bubble was injected after 4, 8 and 12 h of biofilm growth (Fig. 2). For 4 h-old biofilms, the long bubble reduced the surface coverage within patches by nearly two thirds, from 31.6% (±2.4%) to 12.7% (±0.6%). For 8 h-old biofilms, the reduction in surface coverage caused by the bubble was considerably smaller, from 44.6% (±0.9%) to 38.7% (±2.5%), while for 12 h-old biofilms, the reduction was essentially negligible, from 49.5% (±2.3%) to 47% (±2.4%), and the bubble produced no visible change of the biofilm structure. This age-based trend is supported by an analysis of the mean distance between individual cells in the biofilm before and after the passage of the bubble. The rearrangement of the 8 h-old biofilm is evident from the imaging (Fig. 2b and e) and is further supported by a considerable change in the fractal dimension of the cell distribution, from 1.92 (±0.08) to 1.66 (±0.08), while there is no change in the fractal dimension for the 12 h-biofilm (Fig. 2g). Taken together, these measurements indicate that a long air bubble largely removed 4 h-old biofilms, primarily rearranged cells in 8 h-old biofilms without detaching them, and had little effect on 12 h-old biofilms.

**Fig. 3:**
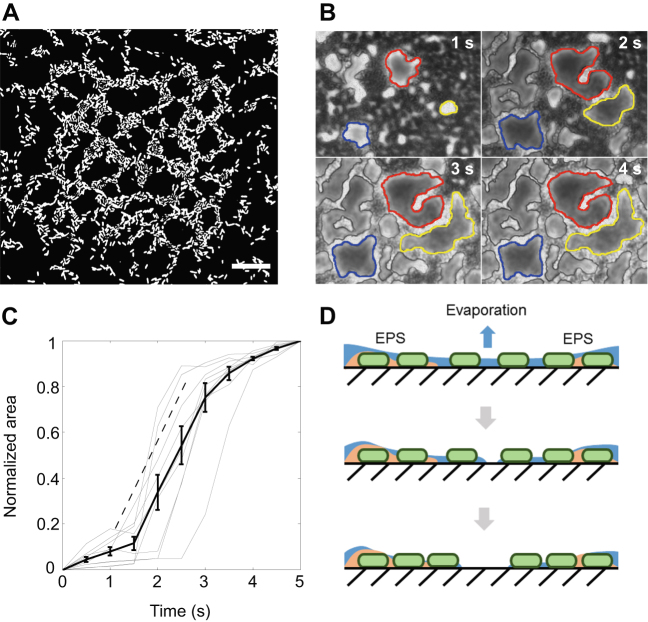
Biofilm holes and hole formation dynamics. **a** Close-up view (×60 objective) of the bacterial distribution in an originally 100^2^ µm^2^ biofilm patch, after the passage of an air bubble. Scale bar, 20 µm. **b** Zoomed-in view of the dynamics of hole formation at 1 s intervals. The boundaries of three holes are identified with *red*, *blue* and *yellow* at each of the four time points. Scale bar, 10 µm. **c** Time course of the area of the nine holes in the image shown in (**b**) (*faint gray lines*) along with their average (*thick black line*). The area of each hole was normalized by its maximum value. The *dashed line* denotes a linear slope. Error bars correspond to the standard error of the mean. **d** Schematic of bacterial repositioning by the action of the evaporating thin liquid layer

Imaging of the hole formation dynamics at high temporal resolution (50 frames s^−1^) revealed that the hole pattern resulted from the rupturing of the residual thin liquid film between the channel surface and the bubble at discrete locations. The ensuing movement of the contact line scraped bacteria outwards from the holes, to form “bacterial levees” between adjacent holes (Fig. 3; Supplementary Video 1). A long air bubble traveling over a solid surface remains separated from it by a thin layer of liquid, whose thickness depends on the capillary number, Ca = *µU*/*σ*, which measures the relative importance of viscous forces and capillary forces. Here, *µ* is the dynamic viscosity of the liquid, *U* is the bubble velocity, and *σ* is the interfacial tension between liquid and air. In our experiments, the bubble traveled at *U* = 250 µm s^−1^ in culture medium contaminated by the presence of bacteria and EPS on the walls of the channel; in these conditions, we estimated *µ* = 10^−3^ Pa s and *σ* = 25–50 × 10^−3^ N m^−1^ (*see* ref. 19), resulting in Ca = (5–10) × 10^−6^. For Ca << 1 the thickness of the liquid film, *h*, follows Bretherton's law,^20^ *h*/*H* ~ Ca^2/3^, where *H* = 50 µm is the height of the microchannel and the constant of proportionality is on the order of unity. This results in an estimated liquid film thickness of *h* ≤ 0.1 µm. Thus, the bubble would create on a flat surface a liquid film that is thinner than the thickness of a bacterium (~1 µm) which would then quickly rupture through an evaporation-driven instability. An evaporation rate of 5 × 10^−5 ^kg m^−2^ s^−1^, determined assuming room temperature and 50% relative humidity (RH), indicates that a 0.1 µm thick water film evaporates in ~2 s (*see* ref. 21). A sensitivity analysis on RH, which is unfortunately unknown in our experiments, reveals that this conclusion is robust for even large variations in RH (for RH between 10 and 90%, the evaporation rate ranges from 10^−5^ to 10^−4 ^kg m^−2^ s^−1^ and the evaporation time from 1 s to 4 s). The evaporation time is thus substantially smaller than the time taken by the entire bubble to pass over any given point on the bacterial film (~10 s; the bubble’s length divided by its speed), indicating that the film can fully evaporate before the bubble has passed.

The evaporation timescale is consistent with the dynamics of hole opening (Fig. 3; Supplementary Video 1), suggesting that—by rapidly thinning the liquid layer—evaporation can lead to the deformation of its free surface in the voids between bacteria and to its ultimate rupture. The resulting three-phase contact line ruptures and moves radially outward from the point of rupture to minimize surface energy. This process is akin to “confined dewetting lithography”, where the thinning and rupture of a liquid film is used to arrange surface-residing colloidal particles into defined patterns.^22^ Because bacteria are thicker than the liquid film, they protrude above the air-liquid interface of the dewetting hole’s receding contact line: the resulting capillary force can dislodge individual cells, which are transported outwards by the receding contact line, until the latter becomes pinned when the accumulated bacteria form a levee between the opening hole and an adjacent hole (Fig. 3b).

An analysis of the time evolution of this process shows that the dynamics of hole formation is rather consistent among different holes within a single patch (Fig. 3c, d) and is characterized by a 3-step process: (i) a slow, initial opening of the hole, followed by (ii) a rapid, linear growth in time, and concluded by (iii) a slow, final growth. The overall process takes approximately 5 s and is therefore consistent with evaporation being the driver of the liquid film’s rupture and with an estimated film thickness of about 0.1 µm; this also means that van der Waals forces—significant only for film thicknesses below 10 nm^23^—can be safely neglected. Although the evaporation of the thin liquid layer occurs regardless of the density of bacteria on the surface, this mechanism only works at intermediate adhesion strengths (8 h-old biofilm on the hydrophobic patch), whereas for younger biofilms, cells are swept away, and for older biofilms, the capillary force is insufficient to scrape cells across the surface. The critical role played by surface tension forces in the formation of holes is confirmed by experiments in which we used high flow rates (up to 100 µl min^−1^), albeit for a short time, and did not observe any spatial re-arrangement of the bacteria as in the case of the passage of an air bubble. This result also shows that shear stress alone was not sufficient in our case to remove attached cells from the surface. Taken together, these results illustrate how the effect of an external insult depends strongly on the stage of biofilm development (here characterized by 1–2 layers of cells in the early stages^24^), and that small variations in biofilm age (a few hours) for early-stage biofilms can have profound effects on their disruption by mechanical insults.

The “age” of the biofilm is not, of course, an absolute quantity, but rather depends on how fast bacteria attach to a substrate and develop micro-colonies. In our experiments, the formation of a biofilm on the hydrophilic substrate outside the patches occurs at a slower rate than on the hydrophobic patches: after 8 h the density of bacteria on the hydrophilic substrate is still relatively low and the capillary effect of the residual films after the passage of the air bubble is similar to the one observed for biofilms on the patches after 4 h (Fig. 2 and Supplementary Fig. S5; Supplementary Video 1). In addition, this result also shows that the detachment and spatial re-arrangement of cells on the surface is largely independent of the specific chemical nature (i.e., hydrophobic vs. hydrophilic) of the substrate and therefore is likely to be a more general phenomenon that may also occur on many natural surfaces.

**Fig. 4:**
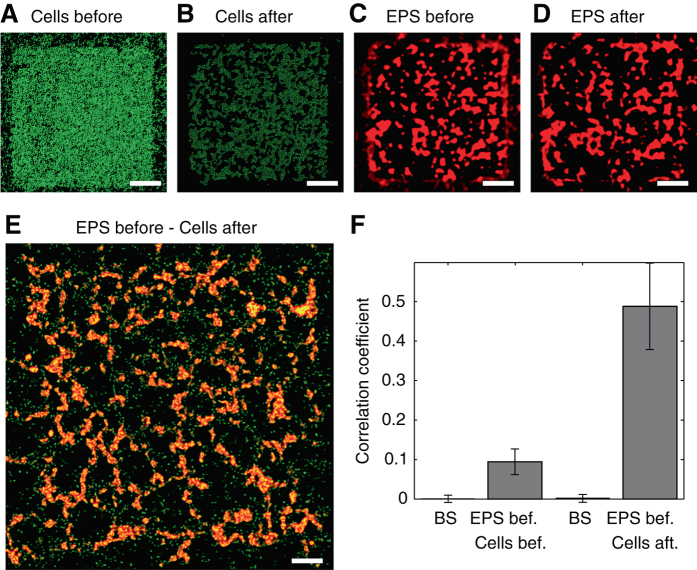
The hole pattern correlates with the EPS distribution. **a**, **b** Distribution of fluorescently labeled *P. aeruginosa* after 8 h of biofilm growth, before (**a**) and after (**b**) the passage of an air bubble. **c**, **d** Distribution of EPS, fluorescently labeled using lectins (Methods), at the same times and position as **a**, **b**. Scale bars, 100 µm. **e** Overlay of the EPS distribution before the passage of the bubble (*red*) and the bacteria distribution after the passage of the bubble (*green*). Data are from a different experiment than **a**–**d**. Scale bar, 50 µm. **f** Normalized cross-correlation between the EPS distribution before the passage of the bubble and the cell distribution before (second bar from left) and after (fourth bar from left) the passage of the bubble. Error bars correspond to the standard error of the mean over three replicate experiments. The first and third bars represent results from a bootstrapping analysis (BS), where the EPS distribution before the air bubble is correlated with random permutations of the cell distribution before (*first bar*) and after (*third bar*) the passage of the bubble. Note the lack of correlation in these controls

A correlation analysis shows that the spatial distribution of EPS is the primary determinant of the local strength of adhesion, strongly suggesting that it is therefore responsible for the observed spatial patterns (Fig. 4). To quantify the distribution of EPS, we injected fluorescently labeled lectins^25^ (wheat germ agglutinin (WGA) conjugated with tetramethyl rhodamine isothicyanate (TRITC)) in the microchannel at different times during the biofilm’s growth. Surprisingly, we found that, although bacteria quickly covered the surface of each patch so that after 8 h of growth the cell distribution is nearly uniform (Figs 2b and 4a), the distribution of EPS remained highly heterogeneous (Fig. 4c). Most importantly, this EPS heterogeneity is what dictates the appearance of the holes. Indeed, we found a significant correlation (Fig. 4e) between the pre-air-bubble EPS distribution (Fig. 4c) and the post-air-bubble cell distribution (Fig. 4b), and this correlation is 5-fold higher than the correlation between the pre-air-bubble EPS distribution and the pre-air-bubble cell distribution (Fig. 4f). Furthermore, we found that the passage of the bubble had a negligible effect on the distribution of EPS on the surface (Fig. 4c, d). These results reveal that holes in the biofilm structure formed where there was the least amount of EPS before the passage of the bubble, and that cells from regions of low EPS concentration were scraped into regions of high EPS concentration, where they—together with the EPS—formed levees that prevented further hole expansion (Fig. 3d).

The observed disruption pattern caused by air bubbles highlights the microscale heterogeneity intrinsic in the biofilm’s organization, likely reflecting microscale spatial variability in EPS production and/or surface colonization times. This view is inline with recent observations of the role of EPS in guiding the formation of micro-colonies.^26^ Also inline with those earlier findings is the observed characteristic length-scale of the disruption pattern (~10 μm). However, the results reported here add a new dimension to our understanding of biofilm disruption because they demonstrate that an environment in which a surface is initially uniformly colonized—which is different from the trail networks driven by twitching motility^27^—can in fact hide an underlying structure that controls the effect of a mechanical insult on surface colonization. Thus, this work supports the view that the locations where more matrix is initially deposited are those where the biofilm is strongest and represent microscale strongholds that preferentially resist mechanical insult. Here, heterogeneity in the EPS distribution causes a fraction of the cells to have the highest chances of retaining their position on the surface while others are dislodged or rearranged.

## Conclusions

We have demonstrated that mechanical insults can result in partial removal of biofilms, depending on biofilm age, with the emergence of characteristically heterogeneous patterns. Correlation of these patterns with the spatial distribution of EPS abundance suggests that microscale heterogeneity in initial colonization and in later EPS production can have major consequences on the local strength of adhesion of biofilm cells, and ultimately result in a major rearrangement in the biofilm’s organization after a mechanical insult. These findings point to the role of biofilm age and the associated heterogeneity in adhesion strength on biofilm removal, and should be considered in the design of shear-based or bubble-based removal strategies.

Biofilms are often assumed to be homogeneous and this view has affected biofilm models. However, the results presented here show that the time history of biofilm formation—and the associated spatial heterogeneity in the distribution of EPS and thus of the mechanical properties of a biofilm—is not only important for its initial formation dynamics, but is retained as memory in the system also for later macroscopic processes such as biofilm disruption by mechanical forces. This microscale heterogeneity implies the existence of strong differences in the mechanical microenvironment of cells in a biofilm, but likely also in the chemical microenvironment and possibly in the cells’ physiological characteristics. One example could be the formation of biofilms in porous media and groundwater systems in which it is common to have low flow rates and a multiphase flow. These micro-gradients may have multiple repercussions on biofilm dynamics, open new ecological niches, foster phenotypic heterogeneity and influence susceptibility to antimicrobials.

## Methods

### Materials

OTS [CH_3_(CH_2_)_17_SiCl_3_] (97%), hexane (anhydrous, 99%), and fluorescein isothiocyanate-bovine serum albumin (FITC-BSA) and TRITC-WGA were purchased from Sigma-Aldrich (St. Louis, MO, USA). Polydimethylsiloxane (PDMS, Sylgard 184) was purchased from Dow Corning.

### Bacterial strain, growth conditions and device operation

The wild-type strain of *P. aeruginosa* PA01 was used for this study (courtesy of George O’Toole, Dartmouth University). For the preparation of the cell culture, cells from freezer stocks were inoculated in LB medium (10 g L^−1^ NaCl, 5 g L^−1^ yeast extract, 10 g L^−1^ tryptone) at 30 °C under shaking (150 rpm). Cells were resuspended in fresh LB medium and incubated at 37 °C under shaking (180 rpm) up to OD_600_ = 0.2, corresponding to early exponential phase. An aliquot of this cell suspension was injected and left for 1 h in a 4 mm wide, 50 µm tall microfluidic channel to allow cells to adhere to surfaces. Thereafter, continuous injection of M63 minimal medium (which contains salts that supply nitrogen, phosphorus, and trace metals) and 0.5% glucose at a constant flow rate of 3 µl min^−1^ began, and this flow rate was maintained for 4, 8 or 12 h, respectively, to produce biofilms of different ages. This flow rate corresponds to an average flow velocity of 250 µm s^−1^ and a shear rate at the bottom surface of ~30 s^−1^.

### Air bubble generation

To introduce controlled air bubbless in the microchannel, we used a three-way valve: one inlet for the bacterial solution (used for initial injection), one inlet for the bacteria-free M63 medium (used for 4 h, 8 h or 12 h), and one inlet for atmospheric air to generate air bubbles. The latter were introduced in the channel through the use of a syringe pump and valve.

### Generation of patterned hydrophobic coatings

PDMS stamps were fabricated by curing the prepolymer on silicon masters patterned with SU-8 photoresist (SU-8 2050, MicroChem, MA, USA) using conventional soft photolithography. The masters used for patterning had recessed features, which resulted in PDMS replicas with protruding features.^28^ To assist in removal of cured PDMS from the SU-8 masters, the latter were silanized overnight by exposure to the vapor of 1,1,2,2,-tetrahydrooctyl-1-trichlorosilane, CF_3_(CF_2_)_6_(CH_2_)_2_SiCl_3_. To cure the PDMS prepolymer, a mixture of 10:1 silicon elastomer and the curing agent was poured onto the master and held at 65 °C for 2 h. The PDMS replica was then peeled from the silicon master. Hydrophobic patterns of OTS on the glass substrate were made by using these PDMS stamps for microcontact printing (Supplementary Fig. S1). The PDMS stamp was inked with a 2 mM hexane solution of OTS and dried in air for 5 min, then placed in contact with the glass substrate at room temperature for 30 s. The stamp was carefully removed and the substrate was rinsed with 2-propanol (IPA) and DI, then dried. Because the trichlorosilane reagents are sensitive to the water content and temperature of the printing environment, the RH and the temperature of the room were kept constant at ~50–55% and ~22–24 °C, respectively. We tested the performance of the microstamping process by assessing the presence and integrity of OTS patches using a small amount of bovine serum albumin labeled with fluorescein isothiocyanate (FITC-BSA, 1 µg mL^−1^ in Phosphate-buffered saline). Epifluorescence images of FITC-BSA adsorbed on the surface reveal (Supplementary Fig. S2) strongly selective adsorption on the hydrophobic patches.

### Microscopy and image analysis

Epifluorescence microscopy imaging was performed using an inverted microscope (Nikon TE-2000E) equipped with green fluorescent protein and red fluorescent protein filter sets. Images were acquired with ×40 and ×60 objectives and an Andor iXon CCD camera (50 frames s^−1^) cooled to −65 °C. Image analysis was performed using built-in plugins of the ImageJ software (http://rsbweb.nih.gov/ij/). Surface coverage, porosity and average hole radius of the biofilms were calculated using standard image processing techniques. The fractal dimension was computed using the box-counting method,^29^ as the slope of the linear fit of ln(*N*) against ln(1/*s*), where *N* is the number of boxes that cover the bacterial patches and *s* is the box size.

## Electronic supplementary material

Supplementary Information

Supplementary Video
