# Supplementary material for: Biofilm disruption by an air bubble reveals heterogeneous age-dependent detachment patterns dictated by initial extracellular matrix distribution
Source: NPJ Biofilms Microbiomes. 2017 Mar 3;3:6. doi: 10.1038/s41522-017-0014-5 (PMC5460265; doi:10.1038/s41522-017-0014-5)
Supplement: Supplementary file 1 — Supplementary Information [file 41522_2017_14_MOESM1_ESM.pdf]

Supplementary Figures

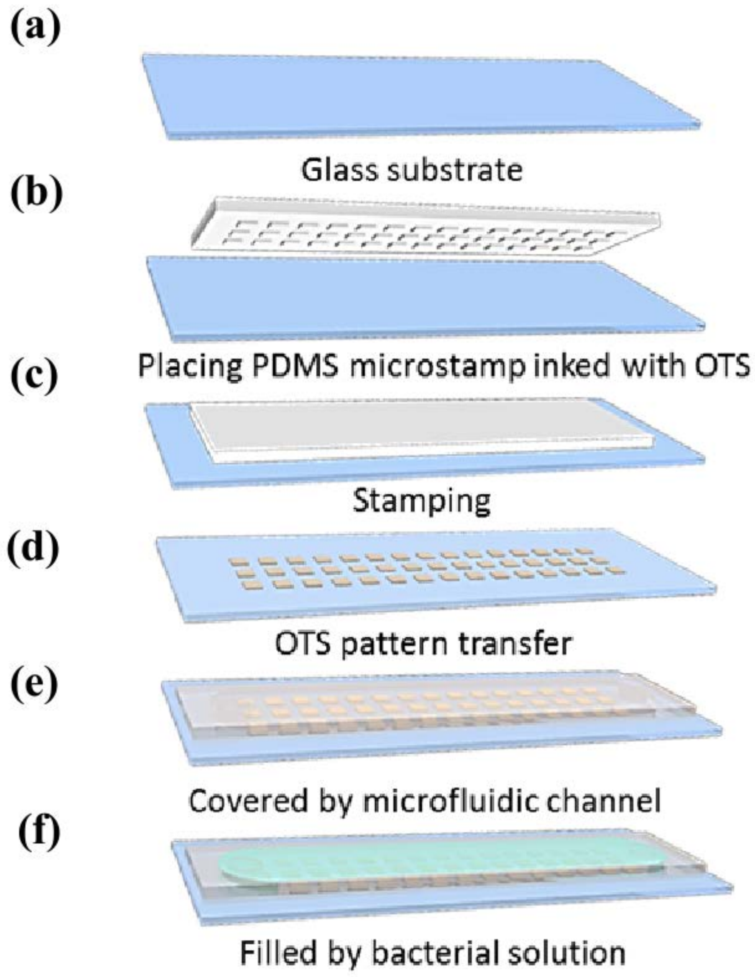

Figure S1. Schematic of the microcontact printing technique used for producing hydrophobic (here, OTS) patches onto a glass substrate.

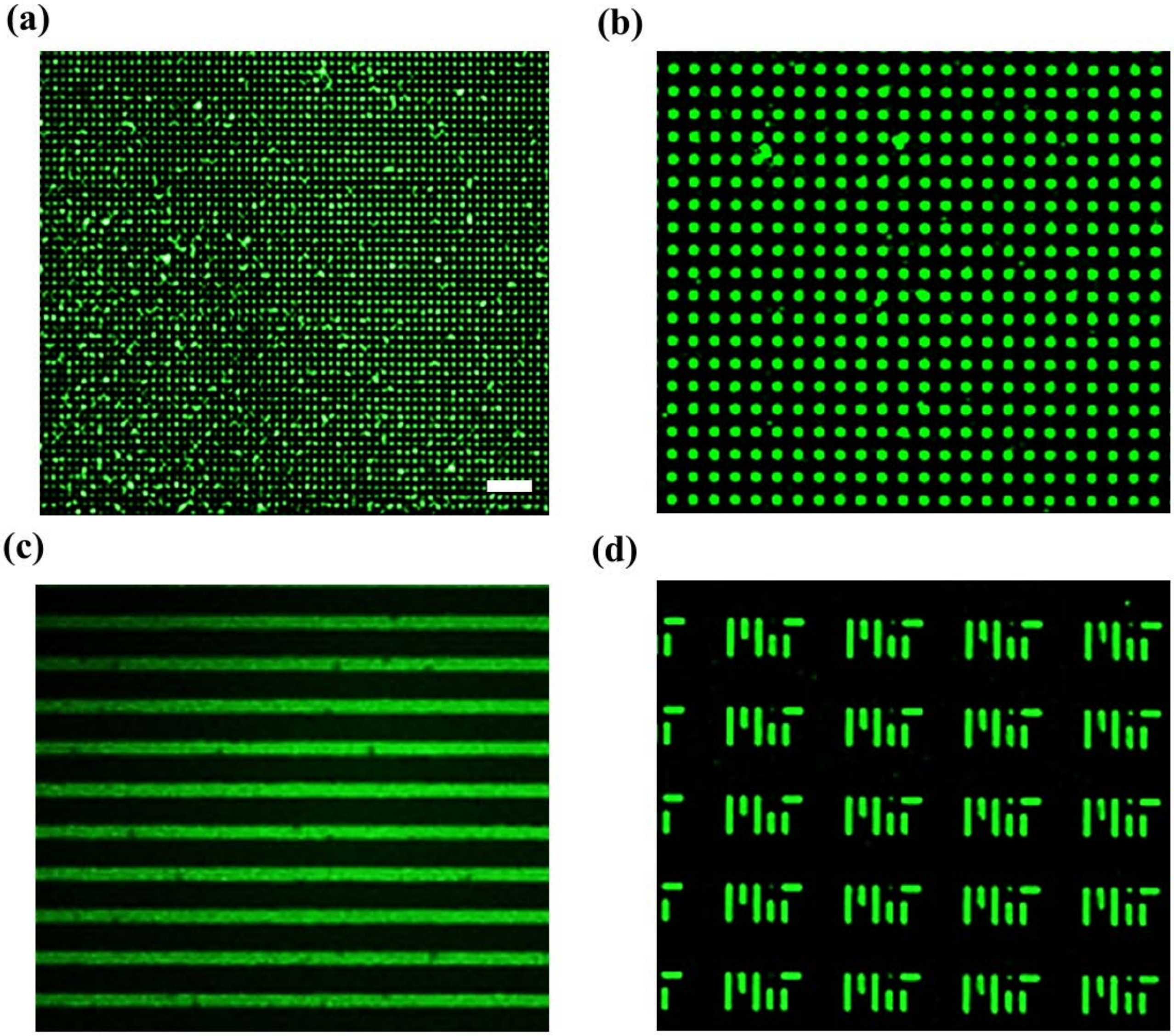

Figure S2. Fluorescent images of FITC-labeled bovin serum albumin (BSA) absorbed onto the hydrophobic patches, demonstrating the specificity of the microcontact printing technique. Shown are different types of patterns – 20 and 50 µm square patches (a-b), 100 µm-wide lines, and the MIT logo – illustrating the flexibility of the technique. In this work, square patterns were used to create localized biofilm patches. Scale bar, 300 µm.

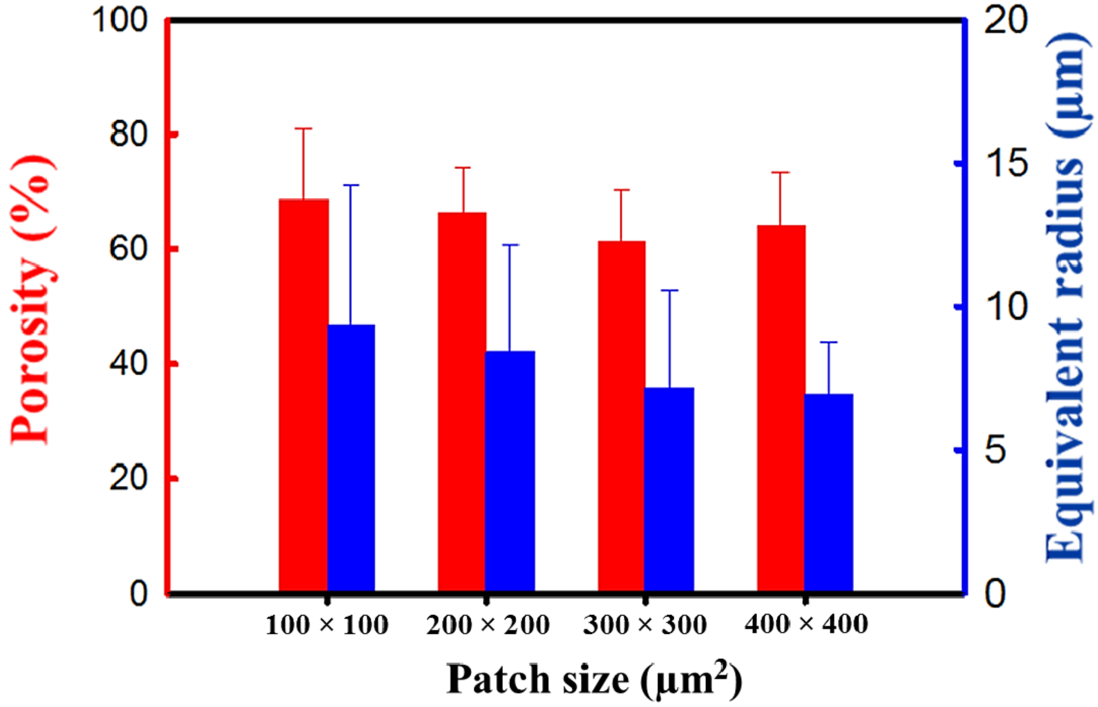

Figure S3. Porosity (red, left axis) and equivalent hole radius (blue, right axis) for experiments with patches of different sizes (*x* axis). The equivalent hole radius was determined by measuring the area of the hole through image analysis and determining the radius of the circle with equal area. Error bars represent standard deviations.

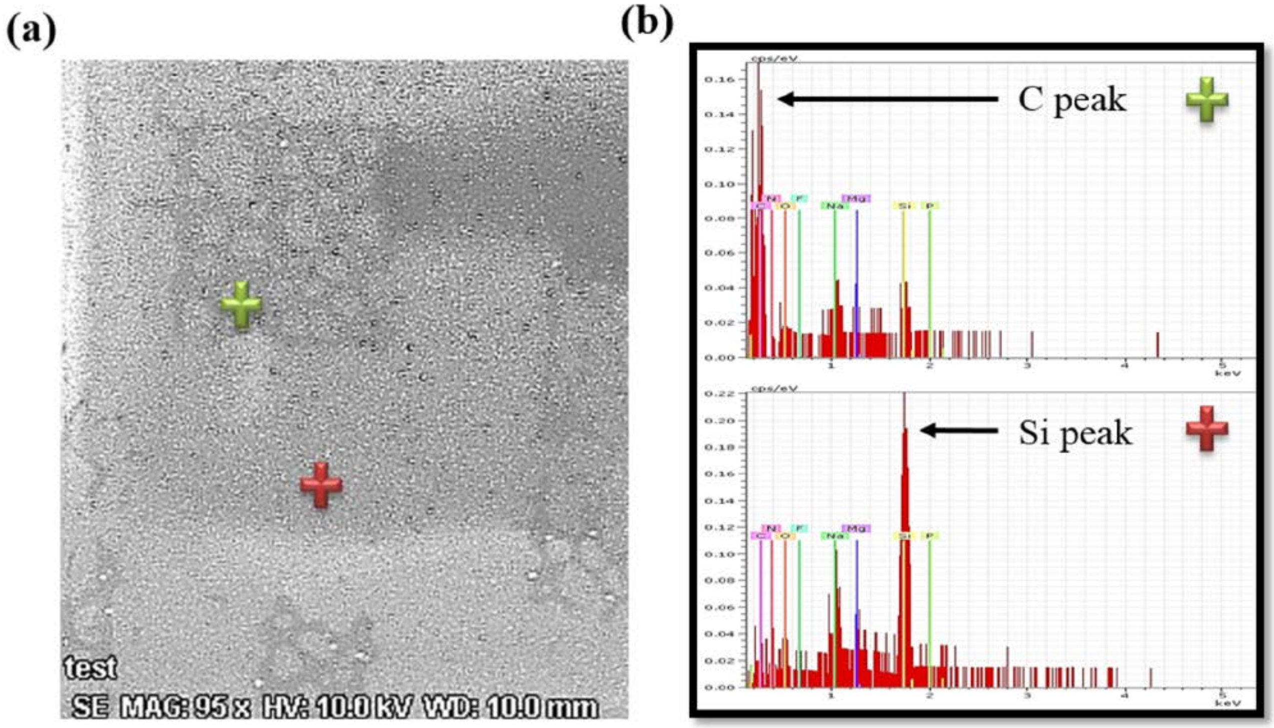

Figure S4. (a) Secondary electron micrographs with spot EDX analysis of *P. aeruginosa* biofilm patches. EDX is an analytical technique used for the chemical characterization of a sample. Briefly, the interaction between X-ray excitation and the sample surface is used to determine the surface composition. Carbon–containing substances are dominant in the regions corresponding to biofilm holes (green cross in panel a; top plot in panel b), whereas silicon-containing substances are dominant in the uncoated glass regions (bottom plot of panel b). The lack of a Si peak in the biofilm hole region indicates that this region is still covered by OTS or by cells, and that the OTS coating has thus not been removed by the passage of the air plug.

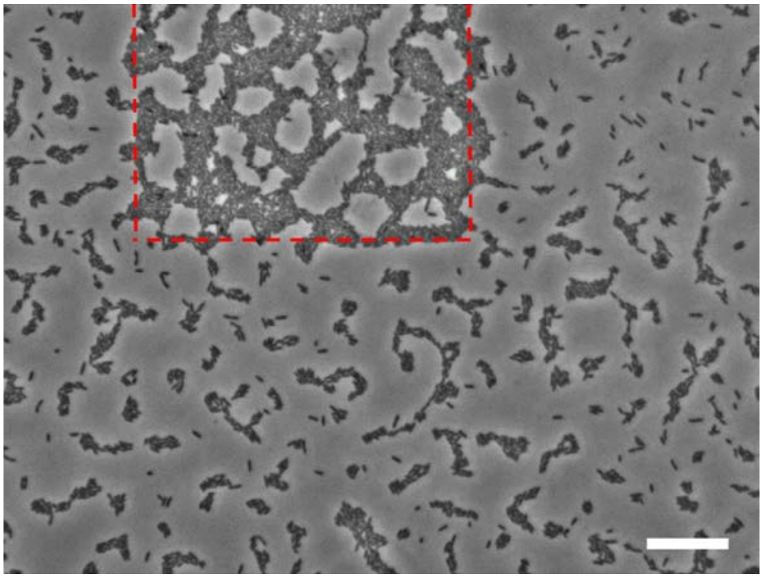

Figure S5. Bacterial distribution around the hydrophobic patch (highlighted by the red dotted line) after the passage of an air plug at 8 hours of biofilm growth. Scale bar, 50 µm.

Movie S1. Time-lapse imaging of the formation of holes in a 400 µm-size biofilm patch after the passage of an air bubble. The frame rate is 10 fps, the total duration time is 5 s.
